# The first joint ESGAR/ ESPR consensus statement on the technical performance of cross-sectional small bowel and colonic imaging

**DOI:** 10.1007/s00330-016-4615-9

**Published:** 2016-10-18

**Authors:** S. A. Taylor, F. Avni, C. G. Cronin, C. Hoeffel, S. H. Kim, A. Laghi, M. Napolitano, P. Petit, J. Rimola, D. J. Tolan, M. R. Torkzad, M. Zappa, G. Bhatnagar, C. A. J Puylaert, J. Stoker

**Affiliations:** 1 Centre for Medical Imaging, University College London, 250 Euston Road, London, NW1 2BU UK; 2 Department of Paediatric Imaging, Jeanne de Flandre Hospital, Lille University Hospitals, Av Eugène Avinée, Lille, France; 3 Department of Radiology, Mater Misericordiae University Hospital, Dublin 7, Ireland; 4 Department of Radiology, Hôpital Robert Debré, Reims, France; 5 Department of Radiology, Inje University College of Medicine, Haeundae Paik Hospital, Haeundae-ro 875, Haeundae-gu, Busan, 612-030 Korea; 6 Department of Radiological Sciences, Oncology and Pathology, Sapienza University of Rome, I.C.O.T. Hospital, Via Franco Faggiana, 1668, Latina, 04100 Italy; 7 Department of Radiology and Neuroradiology, V. Buzzi Children’s Hospital, 32 Castelvetro Street, Milan, 20154 Italy; 8 Department of Paediatric Radiology, Timone Enfant Hospital, 264 rue St Pierre, 13385 Marseille Cedex 05, France; 9 Radiology Department, Hospital Clínic Barcelona, University of Barcelona, C/Villarroel 170, Catalonia, Barcelona 08036 Spain; 10 St James’s University Hospital, Leeds Teaching Hospitals NHS Trust, England, UK; 11 Department of Radiology, Hôpital Beaujon, AP-HP, Université Paris 7, INSERM CRI U1149, Clichy, France; 12 Department of Radiology, Academic Medical Centre, University of Amsterdam, Post box 22660, 1105 AZ Amsterdam, The Netherlands

**Keywords:** Crohn disease, Small bowel, Computed tomography, Magnetic resonance imaging, Ultrasound

## Abstract

**Objectives:**

To develop guidelines describing a standardised approach to patient preparation and acquisition protocols for magnetic resonance imaging (MRI), computed tomography (CT) and ultrasound (US) of the small bowel and colon, with an emphasis on imaging inflammatory bowel disease.

**Methods:**

An expert consensus committee of 13 members from the European Society of Gastrointestinal and Abdominal Radiology (ESGAR) and European Society of Paediatric Radiology (ESPR) undertook a six-stage modified Delphi process, including a detailed literature review, to create a series of consensus statements concerning patient preparation, imaging hardware and image acquisition protocols.

**Results:**

One hundred and fifty-seven statements were scored for agreement by the panel of which 129 statements (82 %) achieved immediate consensus with a further 19 (12 %) achieving consensus after appropriate modification. Nine (6 %) statements were rejected as consensus could not be reached.

**Conclusions:**

These expert consensus recommendations can be used to help guide cross-sectional radiological practice for imaging the small bowel and colon.

***Key points*:**

• *Cross*-*sectional imaging is increasingly used to evaluate the bowel*

• *Image quality is paramount to achieving high diagnostic accuracy*

• *Guidelines concerning patient preparation and image acquisition protocols are provided*

## Introduction

Increased utilisation of cross-sectional techniques to image the bowel has occurred in recent years, particularly in the context of inflammatory bowel disease. A large body of evidence now supports the use of computed tomography (CT), magnetic resonance imaging (MRI) and ultrasound (US) in this context, and recent expert guidelines recommend cross-sectional imaging as first line in the diagnosis, staging and follow up of inflammatory bowel disease [1]. In common with many radiological tests, image quality is paramount to achieving high diagnostic accuracy, and there is a risk that rapid dissemination can occur without sufficient attention to acquisition protocols. This is particularly pertinent when imaging the bowel, a complex, motile organ where non-diagnostic examinations are a particular risk. For example, adequate bowel distension is important prior to enteric imaging and correct sequence selection is central to high quality MRI [1]. The European Society of Gastrointestinal and Abdominal Radiology (ESGAR) has formed an expert consensus committee to construct detailed guidelines for performing MRI, CT and US when investigating disorders of the small bowel and colon, with an emphasis on inflammatory bowel disease. Representatives from the European Society of Paediatric Radiology (ESPR) also participated, given the widespread use of cross-sectional imaging in the paediatric age group. The specific remit of the committee was to produce up to date guidelines concerning patient preparation and image acquisition protocols for MR enterography (MRE)/CT enterography (CTE), MR enteroclysis/CT enteroclysis and US/hydrosonography (hydroUS). Standards for interpretation and reporting examinations, together with consideration of clinical indications, diagnostic accuracy and advantages and disadvantages of each modality were beyond the remit of the committee and covered by the recent joint European Crohn’s and Colitis Organisation (ECCO)/ESGAR recommendations for imaging in inflammatory bowel disease [1]. This article reports the recommendations of the expert panel.

## Methods

### Expert panel selection

A call for expressions of interest to take part in the process was circulated to all ESGAR members. From those expressing interest, a panel of ten members (including the chair) were invited based on publication record in the field and geographical location to ensure, as far as possible, appropriate representation across the ESGAR membership. The committee was then supplemented by three members chosen by the ESPR for all paediatric considerations, to give a total of 13 individuals. Two research fellows were added to help with the literature review and document construction, but they did not take part in committee consensus voting.

### Consensus process

**Fig. 1:**
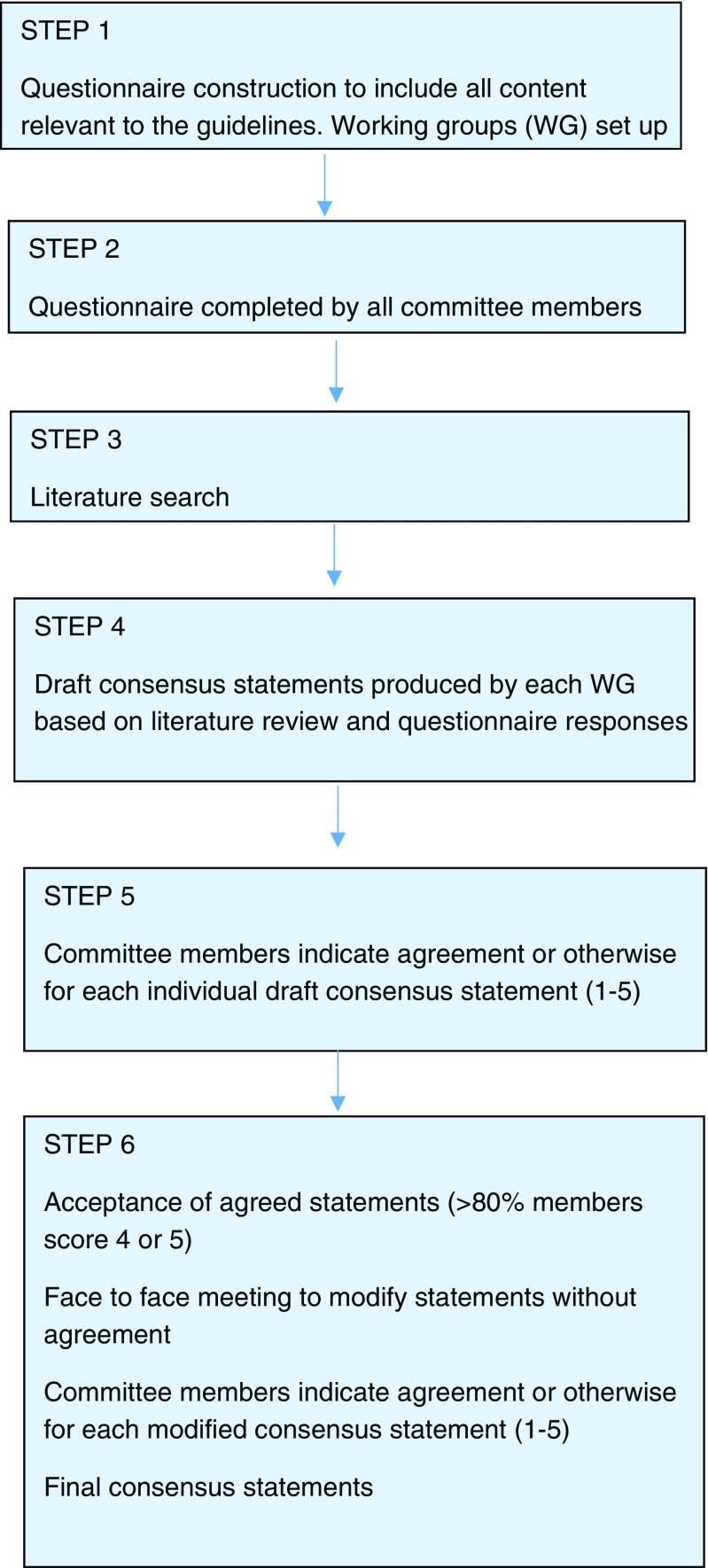
Summary of consensus process

A modified Delphi approach based on the RAND-UCLA appropriateness method was utilised [2], encompassing a detailed literature review and collective judgement of experts, including electronic and face to face discussion [3]. A summary of the process is given in Fig. 1

#### Step 1—Questionnaire construction

The consensus committee met for a face to face discussion to define the scope and aims of the process (11 June 2015), and an initial questionnaire including 185 items was drafted by the chair and then circulated electronically to all committee members. Each item consisted of a specific question with an appropriate range of possible responses, including an option for free text. The questionnaire was split into four broad topics: (1) patient preparation for MRE/MR enteroclysis/CTE/CT enteroclysis, (2) MRE/ MR enteroclysis technique and sequence selection, (3) CTE/CT enteroclysis technique, and (4) enteric US patient preparation and technique. Items were duplicated for both adult and paediatric populations as appropriate. The questionnaire was then modified based on feedback from committee members and a final version agreed upon, which defined individual topics requiring consensus statements. The consensus committee was split into four subgroups matching the four broad topics. Members of ESPR formed a fifth subgroup for paediatric considerations throughout the questionnaire.

#### Step 2—Questionnaire completion

The agreed questionnaire was circulated electronically to committee members who completed their responses to each item in order to document initial expert opinion. The responses were summarised centrally.

#### Step 3—Literature search

A radiology research fellow performed a detailed literature search based on the strategy used by Puylaert et al. [4]. Full details of the search are given in Table 1. The fellow reviewed the retrieved abstracts and selected those pertinent to the items in the questionnaire. Queries were resolved by face to face discussion with the committee chair. The final search retrieved a list of 727 publications, which were circulated to all committee members, along with full abstracts. Working groups were at liberty to update the literature search at their discretion.

**Table 1:** Literature search strategy (from Puylaert et al. [4])

**Search details**	Time period: January 1983–December 2015
**Medline search**	
1	Crohn’s disease
2	Crohn [tiab]
3	Inflammatory bowel disease
4	1 OR 2 OR 3
5	Computed tomography
6	CT [tiab]
7	MRI
8	“Magnetic resonance” [All fields] OR (“magnetic” [All fields] AND “resonance” [All fields])
9	Ultrasound
10	5 OR 6 OR 7 OR 8 OR 9
11	4 AND 10
**Embase search**	
1	Crohn’s disease.ab,ti,sh,kw
2	Inflammatory bowel disease.ab,ti,sh,kw
3	1 OR 2
4	Computer Assisted Tomography.ab,ti,sh,kw
5	Exp Computer Assisted Tomography/
6	Nuclear magnetic resonance imaging.ab,ti,sh,kw
7	Exp Nuclear magnetic resonance imaging/
8	Echography.ab,ti,sh,kw
9	Exp echography/
10	4 OR 5 OR 6 OR 7 OR 8 OR 9
11	3 AND 10
**Cochrane search**	
1	Crohn disease [Mesh]
2	Inflammatory bowel disease [Mesh]
3	1 OR 2
4	Diagnostic techniques and procedures [Mesh]
5	3 AND 4

#### Step 4—Draft consensus statements

Within their working groups, committee members drafted consensus statements for each item listed in the questionnaire based on the available literature and expert opinion as appropriate (using the summarised committee responses to the questionnaire). Members were instructed to always base their statements on the retrieved literature wherever possible, and to this end graded the strength of retrieved relevant publications from I (high) to V (low) using the criteria of the Oxford Centre for Evidence Based Medicine [5] during their review process. If no relevant literature was available for a particular item, members used expert opinion to construct the consensus statements. Each working group produced a list of draft consensus statements, a summary of the supporting literature and table of relevant publications with evidence strength grade, which was then circulated to the whole committee.

#### Step 5—Committee voting

All committee members graded their agreement with each draft consensus statement from 1 to 5 according to the following definitions; 1, strongly disagree; 2, somewhat disagree; 3, undecided; 4, somewhat agree; 5, strongly agree. The responses were summarised centrally.

#### Step 6—Construction of final consensus statements

Those statements achieving a score of 4 or 5 by at least 80 % of committee members in step 5 were accepted into the final set of consensus statements. Those not achieving this were re-discussed at a face to face meeting of the committee (6 March 2016) attended by seven members. Those members who were unable to attend contributed via electronic submissions, which were presented by the chair. Statements not achieving consensus were reviewed with reference to the literature summaries produced by each working group, and committee member opinions were sought. The statement was then either modified or deleted if it was clear consensus could not be reached. The list of revised statements was recirculated to the whole committee who graded agreement from 1 to 5 as in step 5. Those statements achieving a score of 4 or 5 by at least 80 % of committee members were added to the final set of consensus statements.

## Results

The current clinical practice of the panel members is summarised in Table 2. The 13 voting committee members were from the UK (2), Sweden (1), France (4), Netherlands (1), Korea (1), Italy (2), Spain (1) and Ireland (1). Eleven out of 13 panels members used 1.5 T for MR and 2 members used 3 T routinely. Nine panel members had access to CT with at least 64 slices and two had access to a 16-slice scanner.

**Table 2:** Current clinical practice of expert committee members (*n* = 13)

	Panel members performing routinely	Mean annual case load
MRE	13	313
MR enteroclysis	4	14
CTE	8	70
CT enteroclysis	4	23
US	5	110

*MRE* MR enterography, *CTE* CT enterography, *US* enteric ultrasound

The final agreed upon questionnaire consisted of 157 items generating individual consensus statements. At the first round of committee voting, 129 statements achieved consensus according to the a priori definition. The remaining 28 statements did not achieve consensus and were modified to produce a set of 19 statements, all of which achieved consensus agreement in the second round of voting. Those statements which could not be modified to reach consensus were deleted and are shown in Table 3. The final set of 148 consensus statements underwent further editing by the committee chair to avoid duplication, leaving a total of 125 final consensus statements shown in Table 4.

**Table 3:** Statements for which consensus could not be reached after attempted modification

Statement
• It is recommended that the minimal volume of oral contrast for dedicated MRE/MR enteroclysis or CTE/CT enteroclysis is 500 ml (*IV)*
• Splitting the oral contrast into two loads and scanning after ingestion of each to improve small bowel distension is not recommended *(V)*
• The optimal field strength for MRE/MR enteroclysis is 1.5 T *(IV)*
• It is recommended to split-the dose of spasmolytics before T2W sequences and before contrast-enhanced T1W sequences *(V)*
• It is recommended to routinely use small bowel motility sequences during MRE *(V)*
• It is recommended to routinely use an axial diffusion-weighted sequence during MRE/MR enteroclysis *(V)*
• It is recommended that if a spasmolytic is used, and hyoscine butylbromide is unavailable/ contraindicated, a second-line agent is employed during CTE/CT enteroclysis
• It is recommended to administer a spasmolytic before MRE in the paediatric population *(V)*
• It is recommended that if CT scanning is used in the paediatric population, no specific preparation is usually required although administration of positive oral contrast could be considered; for example, prior to percutaneous drainage of abscesses *(V)*

Evidence strength (Oxford Centre for Evidence Based Medicine) shown *in parentheses*

**Table 4:** Final list of consensus statements (achieving agreement score 4 or 5 by at least 80 % of committee members) ^a^

**ADULT PATIENTS**
**1. Patient preparation and basic technique**—**MRE**/**MR enteroclysis**/**CTE**/**CT enteroclysis**
*** Patient preparation***—***general***
• It is recommended that routine medications should not be stopped *(V)*
• It is recommended that patients should not eat any solid food for 4-6 h *(V)*
• It is recommended that patients should not drink any fluid for 4-6 h, although non-sparkling water is permissible *(V)*
*** Basic technique***—***MRE and CTE***
• There is no single preferred contrast agent for MRE or CTE. Recommended agents include mannitol (with or without locust bean gum), PEG, sorbitol and lactulose amongst others *(III)*
• The optimal volume of oral contrast is 1,000-1,500 ml *(III)*
• It is recommended that ingestion time of oral contrast without previous major small bowel resection should be 46-60 min *(V)*
• It is recommended that when scanning patients with a stoma, the stoma should be plugged before oral contrast ingestion *(V)*
• It is not recommended that laxative bowel preparation is administered *(V)*
• It is not recommended that a rectal water enema is administered before a routine examination *(V)*
• It is recommended to administer a liquid enema or prolonged oral contrast preparation without laxative for dedicated colonic evaluation during CTE or MRE *(III)*
• It is recommended to use water as the distension agent if a liquid rectal enema is used for dedicated colonic evaluation *(V)*
• It is recommended that the volume of a water rectal enema is based on patient tolerance if used for dedicated colonic evaluation *(V)*
*** Basic technique***—***MR enteroclysis and CT enteroclysis***
• There is no single preferred contrast agent for MR enteroclysis. Recommended agents include mannitol (with or without locust bean gum), PEG, sorbitol and lactulose amongst others *(III)*
• There is no single preferred contrast agent for CT enteroclysis. Recommended agents include mannitol (with or without locust bean gum), PEG, sorbitol, lactulose and water amongst others *(III)*
• Fluoroscopic guidance for NJ tube insertion prior to MR enteroclysis/ CT enteroclysis is mandatory *(V)*
• It is recommended that the NJ tube for MR enteroclysis and CT enteroclysis should be 8-10 F*(V)*
• It is recommended that contrast infusion before MR enteroclysis or CT enteroclysis should be via an automated pump *(V)*
• It is recommended that the rate of contrast infusion before MR enteroclysis or CT enteroclysis should be between 80 and 120 ml/min *(V)*
• MRI fluoroscopic monitoring of small bowel filling during MR enteroclysis is mandatory *(V)*
• It is recommended that enteric contrast progression should be monitored on the MRI table during MR enteroclysis *(V)*
• It is recommended that enteric contrast progression should be monitored on the CT table during CT enteroclysis *(V)*
• The optimal volume of enteric contrast for MR enteroclysis or CT enteroclysis should be based on monitoring using MRI/CT *(V)* *** Positioning***
• It is recommended that patients can be scanned prone or supine during MRE, CTE, MR enteroclysis or CT enteroclysis *(III)*
**2. MRE**/**MR enteroclysis**—**technical considerations and sequences selection**
*** Hardware***
• Both 1.5 and 3 T are adequate field strengths *(II)*
• The use of phased-array coils is mandatory *(V)*
*** Spasmolytic agents***
• It is recommended that spasmolytic agents are administered during MRE and MR enteroclysis *(II)*
• The timing of spasmolytic agent administration should take into account the susceptibility of the applied MRI sequences to motion artefact *(V)*
• The recommended first line spasmolytic agent is i.v. hyoscine butylbromide *(V)*
• The recommended dose of i.v. hyoscine butylbromide is 20 mg *(III)*
• It is recommended to use a second line spasmolytic agent if the first line agent cannot be given *(V)*
• The recommended second line agent is i.v. glucagon *(V)*
• The recommended dose of i.v. glucagon is 1 mg *(V)*
*** Recommended sequences***
• It is recommend to use the following sequences *(V)* a) Axial and coronal fast spin echo (FSE) T2W sequences without fat saturation
b) Axial and coronal steady state free precession gradient echo (SSFP GE) sequences without fat saturation
c) An axial or coronal FSE T2W sequence with fat saturation
d) Non-enhanced coronal T1W sequence with fat saturation followed by contrast-enhanced coronal and axial T1W sequences with fat saturation
e) In patients with known or suspected inflammatory bowel disease, contrast-enhanced sequences should be in the enteric (45 s) or portal venous phase (70 s)
f) In patients with suspected chronic GI bleeding contrast-enhanced sequences is should be in the arterial (30 s), enteric (45 s) or portal venous phase (70 s) phase
• It is recommended that i.v. gadolinium is pump-injected with an infusion rate of 2 ml/s and a dosage of 0.1–0.2 mmol/kg *(V)*
• It is recommended that the maximal slice thickness for FSE T2W and SSFP GE sequences should be 5 mm *(V)*
• It is recommend that FSE T2W sequences may be performed in either 2D or 3D, although 2D is preferred. *(V)*
• It is recommended that the maximal slice thickness for axial and coronal T1W sequences, should be 3 mm *(V)*
• It is recommended that T1W sequences should be performed in 3D *(V)*
*** Optional sequences***
• Optional additional sequences include an additional FSE T2W sequence with fat saturation, axial and coronal SSFP GE sequences with fat saturation, cine motility and diffusion weighted imaging *(V)*
• It is recommended that a free breathing technique is used if diffusion-weighted sequences are performed *(IV)*
• It is recommended that diffusion-weighted sequences should include lower *b* values ranging from 0 or 50 and upper *b* values ranging from 600 to 900 *(IV)*
• It is recommended that the maximal slice thickness for a diffusion-weighted sequence should be 5 mm *(V)*
• Coronal diffusion-weighted sequences are not recommended *(V)*
• Dynamic contrast-enhanced sequences are not mandatory, but may provide additional information in the form of quantitative measurements of contrast enhancement *(V)*
• Magnetization transfer sequences are not recommended *(V)*
*** Scan coverage and duration***
• It is recommended that scan coverage should include at least the small bowel and colon extended to include the perineum *(V)*
• It is recommended that in general the total acquisition time for should be equal to or less than 30 min *(V)*
**3. CTE**/**CT enteroclysis**—**technical considerations**
*** Hardware***
• MDCT with at least 64 slices is optimal *(V)*
• MDCT with 16 slices or more is considered adequate *(V)*
*** Spasmolytic agents***
• The use of a spasmolytic agent during CTE/CT enteroclysis is optional *(V)*
• It is recommended that if a spasmolytic is used the first line agent is i.v. hyoscine butylbromide *(V)*
• The recommended dose of i.v. hyoscine butylbromide is 20 mg *(IV)*
• The recommended second line agent is i.v. glucagon *(V)*
*** Scan acquisition*** *—* ***general***
• It is recommended that a variable tube current is used, according to the tube voltage used and patient body habitus, but should be kept as low as possible *(V)*
• It is recommended to use automatic exposure control *(III)*
• It is recommended that scan coverage should include the whole abdomen and pelvis including the liver *(V)*
• It is recommended that image-based or raw data-based iterative reconstruction is used if available *(III)*
• The use of multiplanar reformats is mandatory *(III)*
• It is recommended that the maximal slice thickness for displaying axial, coronal and sagittal reconstructed images should be 3 mm *(V)*
• It is recommended that CT acquisition should be cranio-caudal *(V)*
• It is recommended that an upper dose exposure limit is defined *(V)*
• It is recommended that the cumulative value of radiation dose should be recorded, especially in patients affected by chronic conditions resulting in repeat CT imaging *(V)*
*** Scan acquisition*** *—* ***known or suspected inflammatory bowel disease***
• It is recommended that either an enteric phase or portal venous phase acquisition is performed *(III)*
• Additional acquisitions including pre-contrast, arterial, and delayed phase (6-7 min) are not recommended *(V)*
• It is recommended that i.v. iodinated contrast should be pump injected at rate of 3-5 ml/s *(V)*
• It is recommended that i.v. iodinated contrast iodine content is within a range of 300-370 mg/ml *(V)*
• It is recommended that the i.v. iodinated contrast iodine dose should be varied according to the patients’ weight at 1.5 ml/kg *(V)*
• It is recommended that the tube voltage should be within a range of 80-120 according to the patient body habitus *(V)*
*** Scan acquisition*** *—* ***suspected underlying GI bleeding***
• Arterial and portal phases acquisitions are mandatory *(V)*
• It is recommended that i.v. iodinated contrast should be pump injected at rate of 3-5 ml/s *(V)*
• It is recommended that the i.v. iodinated contrast iodine dose should be varied according to the patients’ weight *(V)*
• It is recommended that the tube voltage should be within a range of 80-140 according to the patient body habitus *(V)*
**4. Patient preparation and basic technique**—**enteric US**
*** Patient preparation***
• It is recommended that patients should be nil by mouth for solids for 4-6 h *(V)*
• It is recommended patients should not drink any fluid for 4-6 h prior to the procedure, although water is permissible. If examination of the extra enteric organs is performed, patients should be nil by mouth as per standard protocols *(V)*
*** Hardware***
• It is recommended that evaluation with both low and high frequency probes is performed *(III)*
• The optimal probe frequency for high resolution bowel imaging is 8-10 MHz *(V)*
*** Basic technique*** *—* ***enteric US***
• It is not recommended that laxative bowel preparation is administered *(V)*
• It is not recommended that a rectal water enema is administered before a routine examination *(V)*
• Use of an spasmolytic agent is not recommended *(V)*
• It is recommended that for dedicated colonic evaluation, a standard protocol without specific modification is used *(V)*
*** Basic technique*** *—* ***hydrosonography***
• It is not recommended that laxative bowel preparation is administered *(V)*
• The use of a spasmolytic agent is not recommended *(V)*
• There is no single preferred contrast agent for hydosonography. Recommended agents include mannitol (with or without locust bean gum), PEG, sorbitol and lactulose amongst others *(V)*
• It is recommended that the optimal volume of oral contrast for should exceed 500 ml *(V)*
• It is recommended that ingestion time of oral contrast should be 45 min *(V)*
*** Doppler and IV contrast*** *—* ***US and hydrosonography***
• It is recommended to routinely use colour Doppler *(II)*
• The optimal Doppler flow setting is between 1 and 8 cm/s *(V)*
• Routine use of i.v. US contrast agent is not recommended *(V)*
• If i.v. contrast agent is given, the optimal dose of sulphur hexafluoride is 2.4-4.8 ml *(III)*
• If i.v. contrast agent is given, the standard number of boluses of sulphur hexafluoride is 1 *(V)*
• If i.v. contrast agent is given, the maximum dose of sulphur hexafluoride is 4.8 ml *(V)*
• Scanning between 10 and 40 s after administration of sulphur hexafluoride i.v. contrast is mandatory *(IV)*
• Perfusion or time-intensity curves (e.g. ratio max enhancement/baseline) are not recommended *(V)*
• It is recommended that peak enhancement after contrast injection is measured *(V)*
*** Scan coverage***
• It is recommended that formal reporting of enteric US should state whether the extra enteric organs were examined or not *(V)*
**PAEDIATRIC PATIENTS**—**SPECIFIC CONSIDERATIONS**
**1. Patient preparation and basic technique**—**MRE**/**MR enteroclysis**
*** Patient preparation***
• It is recommended that children aged 6-9 should not eat any solid food for 2-4 h *(V)*
• It is recommended that children aged 6-9 should not undergo fluid restriction *(V)*
• It is recommended that children aged over 9 years should not eat any solid food for 4-6 h *(V)*
• It is recommended that children aged over 9 years should not undergo fluid restriction *(V)*
*** Basic technique***—***MRE***
• It is recommended that the optimal volume of oral contrast for MRE or CTE is 20 ml/kg with a maximum up to 25 ml/kg *(V)*
• It is recommended that the use of a spasmolytic agent is optional *(V)*
• The recommended first line spasmolytic agent is i.v. hyoscine butylbromide, if a spasmolytic is used *(V)*
• The recommended dose of hyoscine butylbromide is 0.5 mg/kg i.v. *(V)*
• The recommended second line agent is i.v. glucagon, if a spasmolytic agent is used *(V)*
• The recommended dose of glucagon in the paediatric population is 0.5 mg (<24.9 kg) and 1 mg (>24.9 kg), given as a slow infusion with i.v. saline at an infusion rate at 1 ml/s *(V)*
• The recommended dose of i.v. gadolinium is 0.1 mmol/kg *(V)*
• It is recommended that the total scan duration should equal to or be less than 45 min *(V)*
**2. Patient preparation and basic technique**—**CTE**/**CT enteroclysis**
• Use of CT scanning in children should be limited to exceptional circumstances, when US and/or MRE cannot address the clinical question *(V)*
• It is recommended that if CT scanning is used, only a portal phase from the diaphragm to the pubic symphysis is acquired *(V)*
**3. Patient preparation and basic technique**—**enteric US**
*** Patient preparation***
• It is recommended that children aged 1-9 should not eat any solid food for 2-4 h *(V)*
• It is recommended that children aged 1-9 years should be nil by mouth for carbonated and milk beverages for 2-4 h. Ingestion of still water or non-carbonated fruit juice is recommended *(V)*
• It is recommended that children aged over 9 years should not eat any solid food for 4-6 h *(V)*
• It is recommended that children aged over 9 years should be nil by mouth for carbonated and milk beverages for 4-6 h. Ingestion of still water or non-carbonated fruit juice is recommended *(V)*
*** Basic technique***—***enteric US***
• Use of laxative bowel preparation is not recommended *(V)*
• Additional colonic distension with a rectal water enema is not recommended *(V)*
• It is recommended that for dedicated colonic evaluation, a standard protocol without specific modification is used *(V)*
• Use of a spasmolytic agent is not recommended *(V)*
*** Doppler and IV contrast***
• The use of i.v. US contrast is not recommended *(V)*
*** Scan coverage***
• It is recommended that scan coverage should include an abdominal and pelvic examination, including the liver *(V)*

Evidence strength (Oxford Centre for Evidence Based Medicine) shown *in parentheses*

*MRE* MR enterography, *CTE* CT enterography, *T1W* T1-weighted, *T2W* T2-weighted

^a^4 = somewhat agree, 5 = strongly agree

## Discussion

The committee considered four main topics as the basis for the consensus statements, with an additional section for specific paediatric considerations.

### General patient preparation and basic MRI and CT technique

There is little evidence on optimal patient preparation prior to MRI or CT, and recommendations with regard to periods of nil by mouth for solids and fluids were based mainly on expert opinion. Ingestion of sparkling water is not recommended due to the risk of producing intraluminal gas artefacts, particularly during MRI. There is good evidence that the accuracy of MRI is improved by administration of oral contrast, in comparison to unprepared MRI [6]. Many oral contrast agents are described in the literature [7–13], but no strong evidence from patient cohorts supports one particular oral contrast agent over another. A number of agents are therefore recommended, usually with hyperosmolar properties [14] and ingested over 46-60 min prior to the examination [15]. Evidence pertaining to the optimal volume of oral contrast agent is limited, although a study in ten healthy volunteers showed inferior distension quality when the ingested volume of oral contrast agent is below 1,000 ml [12]. There was no consensus as to whether the oral contrast agent should be split into two aliquots prior to ingestion or drunk continuously, and both approaches seem acceptable. Although there is evidence that examinations of reasonable quality can be achieved with as little as 450 ml of oral contrast agent [16], no consensus was reached on the minimum oral contrast load for acceptable MRE or CTE and in clinical practice this is usually judged on a case by case basis. Plugging of a stoma is recommended to improve enteric distension, but there is no direct scientific evidence to support this approach. Similarly, in patients with significant bowel resection, scanning earlier, for example at 30 min, may be advisable.

The recommended choice of enteric contrast agents for enteroclysis examinations in general mirrors those recommended for enterography. However, the use of water during CT enteroclysis was included given the speed of CT acquisition, minimising the detrimental effects of gut absorption seen with longer examination times. The optimal volume of contrast agent for both CT enteroclysis and MR enteroclysis should be based on real time on table monitoring.

The routine use of bowel laxatives and rectal enema is not recommended, although there is good evidence that detection of colonic inflammation with MRI is improved with administration of a water enema in comparison to evaluating the unprepared colon [17, 18]. If dedicated colonic evaluation with MRI is required, it is therefore recommended to prepare the colon using a water enema [19, 20], or with prolonged oral preparation [21].

The use of an automated pump for oral contrast administration during enteroclysis is recommended, although hand injection is an acceptable alternative.

Although there is some data suggesting superior bowel distension is achieved in the prone position [22], there is no strong evidence this improves diagnostic accuracy. While also recognising that some patients have difficulty lying prone, either supine or prone positioning is considered acceptable.

### MRE/MR enteroclysis technical considerations and sequences selection

The use of phased array coils at either 1.5 T or 3 T is recommended. There was no consensus for which field strength was optimal for enteric MRI, with data suggesting high image quality is routinely possible on 3 T as well as good quality 1.5 T [23, 24].

There is some evidence that MRE can achieve high diagnostic accuracy without use of a spasmolytic [25], although other data shows significantly superior distension and with the use of these agents [26], particularly in the proximal small bowel, as well as a beneficial effect on study quality by removing peristalsis. Use of spasmolytic prior to MRE and MR enteroclysis is therefore recommended. The literature suggests both hyoscine butyl bromide and glucagon are acceptable agents with differing properties in terms on speed of onset and duration of effect, although both are most effective when given intravenously [27]. Whilst there is volunteer data suggesting superiority of glucagon in achieving complete aperistalsis [28], there is currently no evidence this translates into improved diagnostic accuracy and based on cost, availability and expert opinion, hyoscine butylbromide is recommended as the first-line spasmlotyic, with glucagon as second line.

There was no consensus as to the optimal timing of spasmolytic administration or whether the dose should be split in an attempt to maintain aperistalsis throughout the duration of the MR examination. It is therefore recommended that spasmolytics should be administered before motion sensitive sequences (typically fast spin echo T2-weighted sequence and post contrast T1-weighted images) and either a single or a split dose are acceptable.

There is no available evidence informing the optimal combination of T2-weighted and steady state free precession gradient echo (SSFP GE) sequences, although nearly all studies in the literature utilise both sequence types. Recommendations were therefore mainly based on expert opinion. The use of post-gadolinium T1-weighted images are recommended with data suggesting increased diagnostic accuracy with their use [29, 30], and utility of bowel wall enhancement in validated disease activity scores [31, 32] . There was no available evidence suggesting a single optimal time for post-gadolinium image acquisition, and the recommended options are based on mainly expert opinion.

There is increasing evidence in support of quantified small bowel motility to improve diagnostic accuracy [33, 34], assess disease activity [35–38] and evaluate treatment response [39]. However, at the current time the panel recommends that cine motility sequences remain optional. Similarly, the use of diffusion-weighted imaging (DWI) is increasing, with data supporting its role for disease identification and activity assessment [40–45], and a potential replacement for i.v. contrast-enhanced sequences [46]. It’s advantage over and above conventional MRI sequences, however, is not yet fully established [47] and it is also considered optional at present. It is acknowledged that DWI may have particular utility in the paediatric population [48], and recommendations regarding image acquisition are given. Magnetisation transfer sequences are promising [49] but with little supportive clinical data they are not currently recommended.

### CTE/CT enteroclysis technical considerations

There is little evidence for the optimal CT platform for performing CTE and CT enteroclysis, but based on expert opinion it is recommended that 16-slice CT is a minimum and 64+ slices is optimal. The use of spasmolytic is optional given the speed of CT image acquisition compared with MRI, and the lack of data demonstrating a benefit of spasmolytic. As for MRI, hyoscine butylbromide is the recommended first line agent if a spasmolytic is used. There was no consensus as to the need for a second-line agent if hyoscine butylbromide cannot be administered, but glucagon may used.

CT scan acquisition in either the enteric phase [50, 51] or portal venous phase [52] is recommended in patients with known or suspected inflammatory bowel disease, with no clear evidence supporting one over the other [53]. For patients with suspected GI bleeding, addition of an arterial phase acquisition is considered mandatory to improve detection of vascular lesions [54, 55].

Recurrent exposure of young patients to ionising radiation from CT is a significant concern. Numerous studies have documented high radiation exposure in the IBD patient population, principally from CT [56–61]. There are clear recommendations to minimise patient dose by optimisation of tube voltage [62] and tube current, together with routine use of iterative reconstruction techniques [50, 52, 63–68] which are increasingly available and capable of producing high image quality. For example, a tube voltage in the range of 80-100 kV can reduce radiation dose and increase contrast resolution [64]. The use of automated tube current modulation is also recommended with good data showing dose reduction with maintained image quality [69, 70]. It is recommended that cumulative radiation dose should be recorded for patients with chronic conditions requiring multiple radiological examinations. CT is not recommended in paediatric practice unless there are no alternatives.

### Patient preparation and basic technique—enteric US

There is little evidence on optimal patient preparation prior to US, and recommendations with regard to the period of nil by mouth for food and liquids were based on mainly on expert opinion. There is no evidence supporting the use of bowel laxatives before US or hydroUS and use is not recommend. Detailed assessment of the colon does not require additional preparation.

Similar to MRE and CTE, a range of oral contrast agents for hydosonography is described in the literature [71–73], with no clear evidence for superiority of one over another. A number of agents are therefore recommend, usually with hyperosmolar properties. There is no specific evidence regarding the optimal volume and ingestion protocol for oral contrast and recommendations are based on expert opinion and mainly mirror those of MRE and CTE.

There is no evidence of benefit from spasmolytic administration prior to enteric US, and diminishing the ability of the practitioner to evaluate real time peristaltic activity in the bowel detracts from the examination. Its use is, therefore, not recommended.

There is good evidence supporting the routine use of colour or power Doppler for disease detection and activity assessment during enteric US and hydosonography [74–79].

The routine use of contrast-enhanced US (CEUS) is currently not recommended. However there are increasing reports describing the utility of qualitative evaluation of bowel wall vascularity patterns in CD using CEUS [80, 81], as well as software defined quantitative metrics from time-intensity curves such as time-to-peak, peak enhancement and area under the curve [82–85]. Promising data for evaluation of CD activity against an endoscopic reference have been reported [84, 86], as well as utility in stricture characterisation [87], detection of postoperative recurrence [88], and treatment response evaluation [89]. Apparent diagnostic benefit over conventional US parameters is also described [85] .

However, there are no defined optimal thresholds for quantitative CEUS that differentiate active from inactive disease, and there are differences in perfusion metrics between US manufacturers. The precise role for CEUS in clinical practice is likely to evolve as the evidence base grows.

If CEUS is performed the recommendations on dosage are made on the most widely reported agent (sulphur hexafluoride), although other agents are available. These are based on manufacturer recommendations in the absence of other evidence.

It is recommended that the practitioner specifically reports if the extra enteric solid organs have been evaluated as part of an US examination focused on the bowel. In some clinical practices, it is expected the examination should evaluate the whole abdomen and pelvis, including solid organs, although targeted enteric US (e.g. for treatment follow-up) is also widely practiced.

### Paediatric patients—specific considerations

Although in general, paediatric practice follows that of adults [90, 91], there are important exceptions.

The use of CT is actively discouraged in the paediatric population given the radiation exposure and should be reserved for situations when neither MRI nor US can resolve the clinical question.

Younger children do not tolerate prolonged fluid and food restriction prior to examinations, and recommendations are made according to the age of the patient, mainly based on expert opinion.

Unlike adult practice, the use of spasmolytic prior to MRE is considered optional in paediatric patients and use is likely dependent on the age of the patient, with older children more likely to tolerate spasmolytic injection. There are data supporting the benefits of glucagon on image quality, at the expense of prolonged imaging time and precipitation of nausea in just under half of paediatric patients [92, 93]. However, high diagnostic accuracy can also be achieved without spasmolytic [94]. The choice of spasmolytic, if used, mirrors adult recommendations with due consideration of the age and weight of the patient.

Recommendations for oral contrast volume and spasmolytic/gadolinium dose are based on weight. There are no specific recommendations as to the use of hydroUS in the paediatric patient as practice is not well developed. If oral contrast is given prior to US, it would seem sensible to follow the recommendations for MRE in the paediatric population.

### Limitations

The modified Delphi process utilised in this document is well established, although for the second face to face meeting, not all committee members could attend, with contributions made electronically. However a representative from each working group was present and all committee members subsequently scored their agreement with each modified statement produced. Wherever possible, recommendations were based on the detailed literature review. However, in many areas there was no available evidence, so recommendations were made based on the combined expert opinion of the panel. This will be influenced by the experience and knowledge of the panel members. To mitigate against potential bias, committee members were selected based on publication record and geographical location, and it was ensured that there was experience in all modalities under consideration across the consensus group. Discussions were moderated by the committee chair who was independent of the working groups within the committee members. Finally, these recommendations are relevant at the time of the consensus process (2015/16). The literature in this field is rapidly expanding, and it is anticipated revised guidelines will be required in the future.

In summary, a modified Delphi approach was successfully utilised to produce set of guidelines to help inform current best clinical practice in cross-sectional small bowel and colonic imaging. For only a small number of topics could an agreed consensus statement not be produced by the committee. Whilst there is a clearly a convergence in the literature as to basic techniques, for many specific questions there is no clear evidence base and statements were based on expert opinion, with an emphasis on clinical practicability. Based on the findings of the committee, particular topics can be viewed as research priorities given their potential impact on clinical practice. Examples include the role of DWI and motility imaging as part of MRI protocols, and more detailed guidance on i.v. gadolinium contrast administration given the generally young age of the imaged patient population, frequent repeat imaging and increasing evidence of possible neuronal retention of gadolinium for some contrast agent classes [95]. The clinical utility of micro-bubble contrast agents in small bowel US requires further clarity together with the impact of further advances in dose reduction techniques during CT examinations.
